# Grappling archaea: ultrastructural analyses of an uncultivated, cold-loving archaeon, and its biofilm

**DOI:** 10.3389/fmicb.2014.00397

**Published:** 2014-08-05

**Authors:** Alexandra K. Perras, Gerhard Wanner, Andreas Klingl, Maximilian Mora, Anna K. Auerbach, Veronika Heinz, Alexander J. Probst, Harald Huber, Reinhard Rachel, Sandra Meck, Christine Moissl-Eichinger

**Affiliations:** ^1^Department of Microbiology and Archaea Center, University of RegensburgRegensburg, Germany; ^2^Department of Biology I, Biozentrum Ludwig Maximilian University of MunichPlanegg-Martinsried, Germany; ^3^Zellbiologie, Philipps-Universität MarburgMarburg, Germany; ^4^LOEWE Research Centre for Synthetic Microbiology (Synmikro)Marbug, Germany

**Keywords:** archaea, biofilm, ultrastructure, hami, EPS, SEM, TEM, microbial interaction

## Abstract

Similarly to Bacteria, Archaea are microorganisms that interact with their surrounding environment in a versatile manner. To date, interactions based on cellular structure and surface appendages have mainly been documented using model systems of cultivable archaea under laboratory conditions. Here, we report on the microbial interactions and ultrastructural features of the uncultivated SM1 Euryarchaeon, which is highly dominant in its biotope. Therefore, biofilm samples taken from the Sippenauer Moor, Germany, were investigated via transmission electron microscopy (TEM; negative staining, thin-sectioning) and scanning electron microscopy (SEM) in order to elucidate the fine structures of the microbial cells and the biofilm itself. The biofilm consisted of small archaeal cocci (0.6 μm diameter), arranged in a regular pattern (1.0–2.0 μm distance from cell to cell), whereas each archaeon was connected to 6 other archaea on average. Extracellular polymeric substances (EPS) were limited to the close vicinity of the archaeal cells, and specific cell surface appendages (hami, Moissl et al., 2005) protruded beyond the EPS matrix enabling microbial interaction by cell-cell contacts among the archaea and between archaea and bacteria. All analyzed hami revealed their previously described architecture of nano-grappling hooks and barb-wire basal structures. Considering the archaeal cell walls, the SM1 Euryarchaea exhibited a double-membrane, which has rarely been reported for members of this phylogenetic domain. Based on these findings, the current generalized picture on archaeal cell walls needs to be revisited, as archaeal cell structures are more complex and sophisticated than previously assumed, particularly when looking into the uncultivated majority.

## Introduction

Understanding the microbial “dark matter” has become one of the driving desires of the scientific community (Rinke et al., 2013). In particular, deep-branching, uncultivated archaea have attracted the interest, being largely unexplored but widespread and likely major drivers of the nutrient cycles in various ecosystems (Cavicchioli et al., 2007). Systems that allow unbiased and direct analyses of uncultivated microorganisms on microscopic and macroscopic levels due to one organism's predominance are extremely rare. However, such systems are of utmost importance to understand the functioning of microorganisms in the environment, their natural cellular composition, their actual metabolic activity and their interactions with the abiotic and biotic environment (Morris et al., 2013).

The majority of microorganisms seems to be uncultivable using standard methods (Amann et al., 1995). The unsatisfying success in this regard might be rooted in the interwoven interactivity of microorganisms in their natural biotope, such as natural ecosystems, or macrobes, such as plants or the human body. The human body itself is colonized by 10–100 times more microbial cells than own cells (Schleifer, 2004). Analyzing the (human) microbiome has become a major scientific focus, benefitting from state-of-the-art, cultivation-independent methods which include next generation sequencing of 16S rRNA genes and –OMICS technologies (Zhang et al., 2010). Altogether, these methods allow first glances at the diversity and function of an entire microbial community, which interacts closely with its host, forming a “superorganism”: the holobiont (Margulis, 1993; Rohwer et al., 2002). It is assumed, that the cooperation of host and microbes represents a unit of selection in evolution and changes in composition and function have severe impact on further development or even next host generations (Zilber-Rosenberg and Rosenberg, 2008). As a consequence, evolution appears to be a coordinated process of entire (microbial) communities, which need to be scientifically addressed as a whole.

The effects of microbial interactions for the different partners can vary. In symbiotic relationships all partners benefit, whereas commensal interaction is beneficial for one partner and not harmful for the other. Parasites, however, strongly affect the fitness of one partner (Moissl-Eichinger and Huber, 2011). A well-documented model system of a bacterial symbiotic interaction is “*Chlorochromatium aggregatum*,” a clearly structured consortium of immobile green sulfur bacteria epibionts and a motile beta-proteobacterium (Müller and Overmann, 2011). This association provides mobility to the epibionts and, in exchange, amino acids and 2-oxoglutarate to the inner partner. Detailed ultrastructural analyses revealed that hair-like filaments protrude from the epibionts and directly interconnect with the central bacterium. The latter connects with the epibionts via periplasmic tubes, which attach to the epibiont's outer membrane (Wanner et al., 2008).

In general, structural analyses of syntrophic and interactive consortia and communities that include an archaeal partner have rarely been reported, and information on the structure of natural archaeal populations in the literature is scarce. A likely syntrophic interaction between two hyperthermophilic archaea was artificially established under laboratory conditions: during co-culture conditions, *Pyrococcus furiosus* attaches to *Methanopyrus kandleri* forming an unusual bi-species biofilm on provided surfaces (“fried-egg colonies”; Schopf et al., 2008). The contact between the two types of archaeal cells is mediated by flagella and possibly by extracellular polymeric substances (EPS). One example for a natural and uncultivated archaeal-archaeal interactive community is the ARMAN (archaeal Richmond Mine acidophilic nanoorganisms) system, where the ARMAN cells interact closely with *Thermoplasmatales* cells leading to a potential nutrient or molecule exchange (Comolli et al., 2009; Baker et al., 2010; see also article in this issue).

A model system for archaeal interspecies relationships is represented by the “intimate association” of *Ignicoccus hospitalis* and its partner *Nanoarchaeum equitans* (Huber et al., 2002; Jahn et al., 2008). The relationship is based on the attachment of *N. equitans* to the outer cellular membrane (OCM) of *I. hospitalis* (Jahn et al., 2004). It has been shown that this obligate dependence on *I. hospitalis* is a consequence of the transfer of membrane lipids, amino acids and probably even ATP from *I. hospitalis* to *N. equitans* (Huber et al., 2012). Other investigations gave evidence for the lateral transfer of genetic material in both directions, during the co-evolution of these two archaeal cells (Podar et al., 2008). While *I. hospitalis* is able to grow in pure culture, *N. equitans* still resists cultivation without its host. This system can be maintained in the laboratory, and since one of the microorganisms is strictly dependent on the other, it actually reflects the interaction of two archaea in the natural biotope, where both species thrive.

Moreover, interactive microbial communities of Bacteria and Archaea are known, such as the anaerobic methane oxidizing (AMO) consortia, consisting of anaerobic, methanotrophic archaea (ANME) in loose association with sulfate reducing bacteria (SRB) of the *Desulfococcus/Desulfosarcina* group (Hoehler et al., 1994; Elvert et al., 1999; Hinrichs et al., 1999; Thiel et al., 1999).

Another bacterial/archaeal consortium was detected in the sulfidic springs of the Sippenauer Moor (SM), a cold (~10°C) swamp area, located in the southeast of Germany. Coccoid archaea, designated as “SM1 Euryarchaeon,” were found to be the major constituents of macroscopically visible whitish pearls, floating in the surface waters of the springs. The outer sheath of these pearls is formed by a sulfur-oxidizing, filamentous bacterial partner (*Thiothrix* sp.; Rudolph et al., 2001; Moissl et al., 2002). The pearls are connected by thin threads, exclusively formed by *Thiothrix* sp. (Moissl et al., 2002), giving the microbial community a “string-of-pearls” like appearance. The SM1 Euryarchaeon was also detected in another, distinct sulfidic setting, the Mühlbacher Schwefelquelle (MSI; nearby Regensburg, Germany), where the string-of-pearls community (SOPC) can be found in a similar microbial composition (Rudolph et al., 2004).

Interestingly, subsequent studies revealed that the MSI-SM1 Euryarchaeon seeks the vicinity to sulfide-oxidizers only in (oxygenated) surface waters, whereas in the deeper, anaerobic subsurface it grows as an almost pure biofilm (Henneberger et al., 2006). Within the biofilm, the MSI-SM1 Euryarchaeon predominates a minor bacterial community, which is mostly composed of sulfate-reducing bacteria (Henneberger et al., 2006; Probst et al., 2013). Since the SM1 Euryarchaeon remains uncultured under laboratory conditions, many features, including its metabolic capability, are yet to be fully understood. The archaeal biofilms are transported with the water flow from the subsurface to the spring outflow, where biomass can be harvested in sufficient quantities for further analyses (Henneberger et al., 2006; Probst et al., 2013). Similar biofilms, mainly consisting of coccoid SM1 Euryarchaeota and a minor fraction of bacteria, were also observed in upwelling, anoxic waters of the SM (Henneberger et al., 2006).

The SM1 Euryarchaeon has revealed extraordinary properties, clearly distinguishing it from the archaeal strains characterized in the literature. Firstly, the SM1 Euryarchaeon is one of a few reported archaea capable of biofilm formation in its natural biotope. Additionally, it is the only archaeon known to clearly dominate a low-temperature biotope: the literature suggests that ecosystems are either dominated by bacteria or mixtures of diverse archaea (i.e., Schrenk et al., 2003, 2004; Koch et al., 2006; Webster and Negri, 2006; Weidler et al., 2008; Briggs et al., 2011; Couradeau et al., 2011; Ionescu et al., 2012). The appearance of the SM1 Euryarchaeon in a variety of ecosystems (Rudolph et al., 2004) and in extremely high density (as almost pure biofilms, “hot spots”) suggests an important role in the subsurface with a vast impact on local biogeochemistry. Thirdly, the SM1 Euryarchaeon carries a novel type of cell surface appendages. Being as thin as pili, these appendages (up to 4 μm long) exhibit barb-wire like prickles (which might function as distance holders in the biofilm) and small nano-hooks at their distal end. These structures were described as “hami” (latin for anchors, hooks; Moissl et al., 2005). So far no comparable microbial or artificial similar structures of similar size have been described. These unique properties of the SM1 Euryarchaeon biofilm have made the ecosystems, the microbial assemblages, and the archaeon itself a model system for studying cold-loving archaea in a natural biotope.

The SM1 euryarchaeal biofilms from the two biotopes SM and MSI were compared in a very recent study via genetic and chemical microbiome profiling, which revealed that both biofilms are different in their bacterial composition and are thus unlikely to originate from one single biotope in the subsurface. The archaea of both biofilms were initially judged to be identical - based on an identical 16S rRNA gene of both populations. However, the SM and MSI cells were different in size, showed strong variations in membrane lipid composition and in their genomic information, and revealed also minor differences in ultrastructure (EPS and hami). Thus, we concluded that the two biofilms are dominated by the same archaeal species, but by two different strains thereof (Probst et al., 2014).

Based on this finding, a deeper ultrastructural investigation of the SM population became warranted, which was conducted in this study. Here, we provide novel insights into the multifarious aspects of the SM1 Euryarchaeon lifestyle from structural biofilm organization and the interactions with the bacterial and archaeal neighbors via its unique cell surface appendages to cell wall architecture.

## Materials and methods

### Sampling and sample processing

Samples for ultrastructural analyses were taken in a cold sulfidic spring in close vicinity to Regensburg, Germany (SM; Rudolph et al., 2001, 2004). Archaeal biofilms were harvested from raw-meshed nets, placed right within the spring outflow (Henneberger et al., 2006). The samples were collected using sterile syringes and transported on ice to the laboratory.

### Ultrastructural analysis

Freshly taken biofilms were fixed in original spring water including 0.1% glutardialdehyde (w/v). Scanning electron microscopy was carried out as described elsewhere (Probst et al., 2014). Samples were examined using a Zeiss Auriga scanning electron microscope operated at 1–2 kV. For TEM, the sample preparation and procedure is described in Probst et al. (2014). Samples were examined using a CM12 transmission electron microscope (FEI Co., Eindhoven, The Netherlands) operated at 120 kV. All images were digitally recorded using a slow-scan charge-coupled device camera that was connected to a computer with TVIPS software (TVIPS GmbH, Gauting, Germany).

## Results

### The SM1 euryarchaeon forms a biofilm with EPS and cell surface appendages

**Figure 1:**
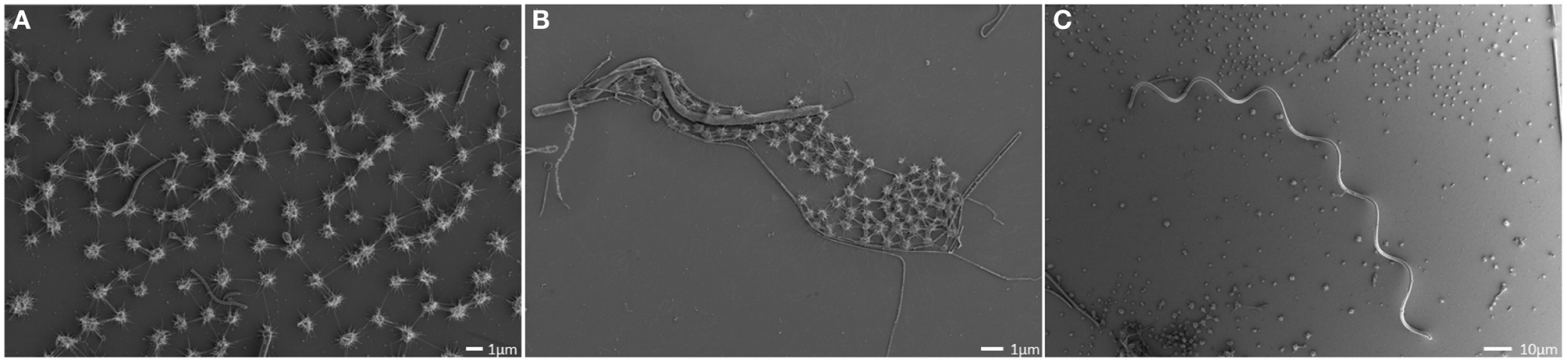
**Scanning electron micrographs of the SM biofilm.** Overview, showing the homogenous archaeal population (small coccoid shaped cells; **A,B**) and large, spiral-shaped bacterium **(C)**.

**Figure 2:**
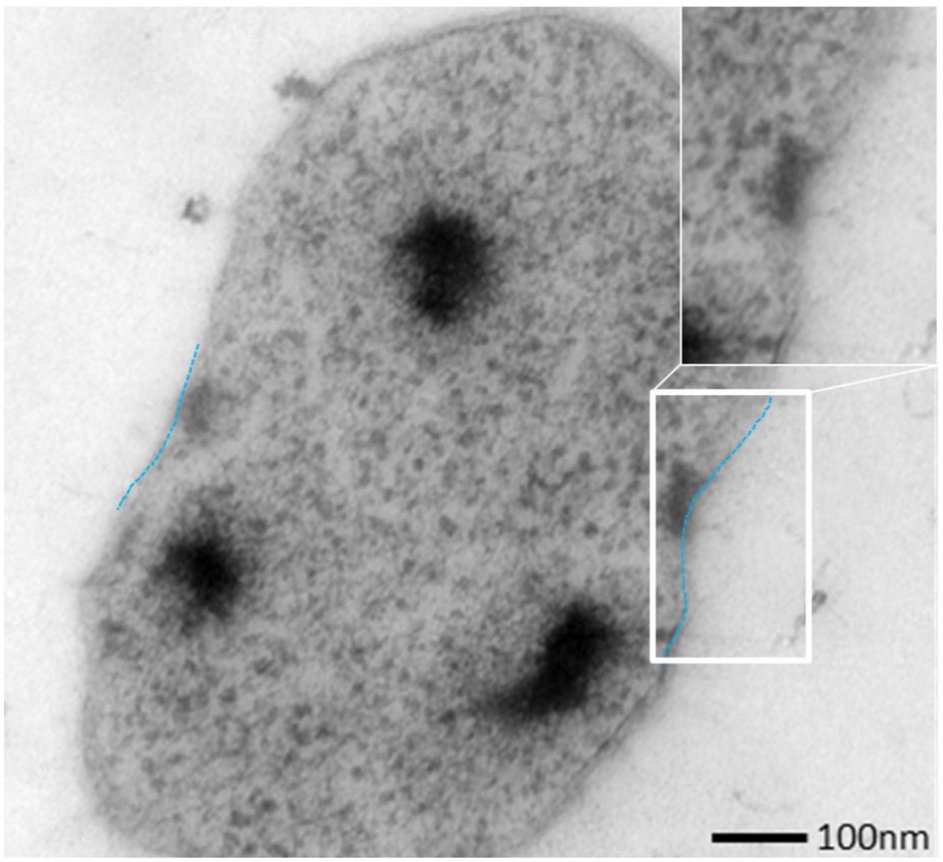
**Ultrathin section of one dividing SM1 coccus with a visible invagination**.

The SM SM1 Euryarchaeon forms a biofilm, which is dominated by a single species. Macroscopically, the biofilm droplets (diameter up to 2 cm) appear milky and viscous, and show strong attachment to various types of surfaces. Using different microscopy techniques, a homogenous cell-population was observed (e.g., Figure 1A). The rare (less than 5%, Probst et al., 2014), mostly unflagellated and unpiliated bacterial cells were embedded within the biofilms and morphologies ranged from short rods, spirilla and cocci to several μm-long filaments (Figures 1B,C). Viruses were not detected in any of the preparations. The archaeal cells were visible as regular cocci, although many cells appeared to be actively dividing at the time point of sampling, with an oval morphology and a clear, central contraction (Figure 2). The average cell diameter of non-dividing cells was determined to be about 0.6 μm (±0.1 μm), corresponding to a cell volume of 0.11 μm^3^ on average (Probst et al., 2014).

**Figure 3:**
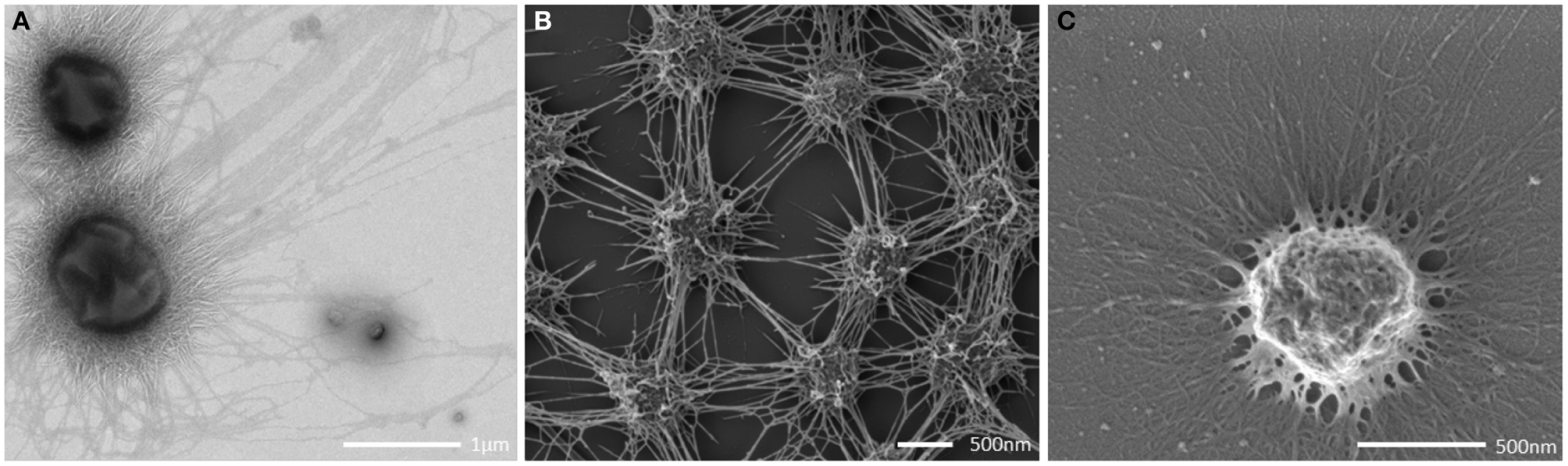
**Transmission electron (A) and scanning electron (B,C) micrographs. (A,B)** show intraspecies contact via the cell appendages (bars: **A**: 500 nm; **B**: 400 nm). **(C)** shows a single coccus embedded in a thick EPS layer.

**Figure 4:**
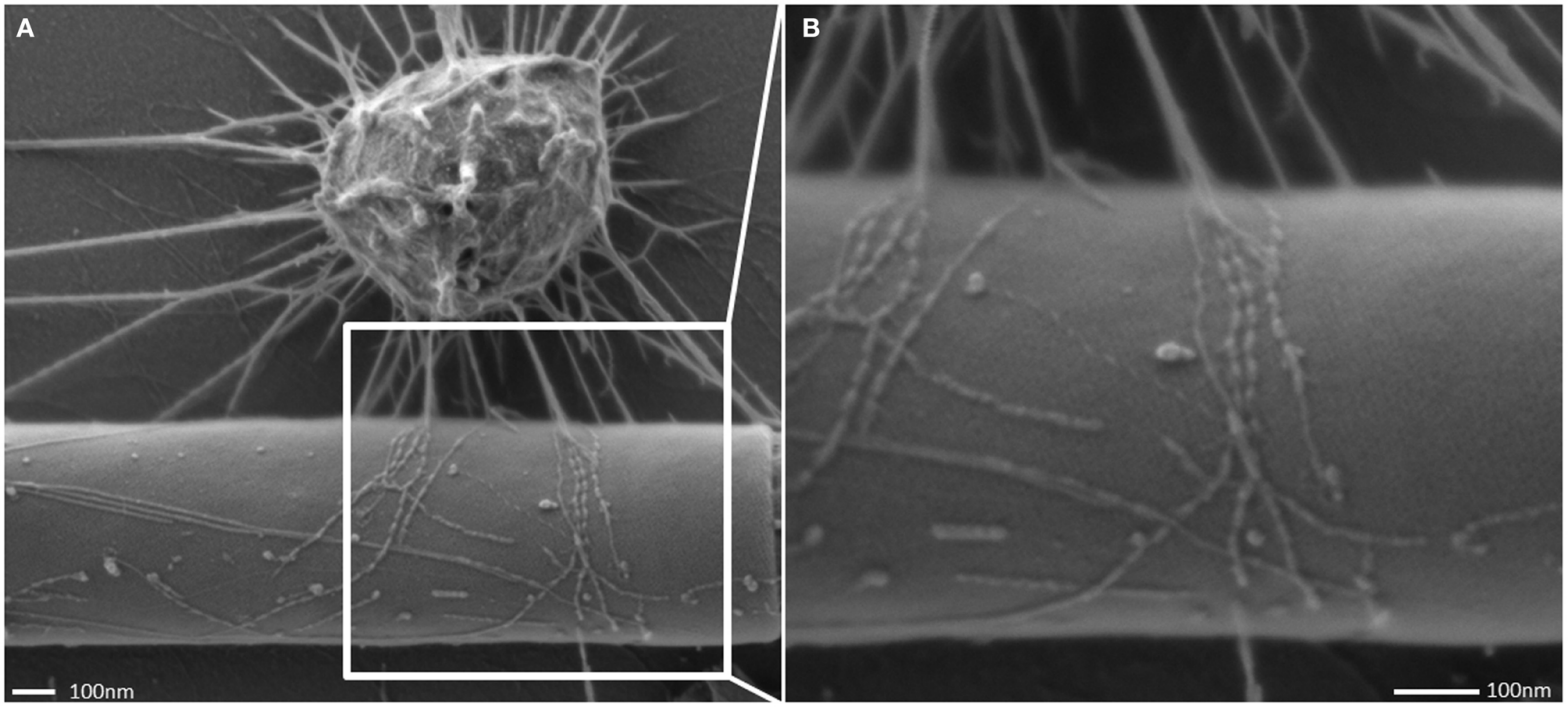
**Scanning electron micrograph of the cell appendages: “hami.”** Hami attaching to a filamentous bacterium **(A)** and close up view **(B)**.

**Figure 5:**
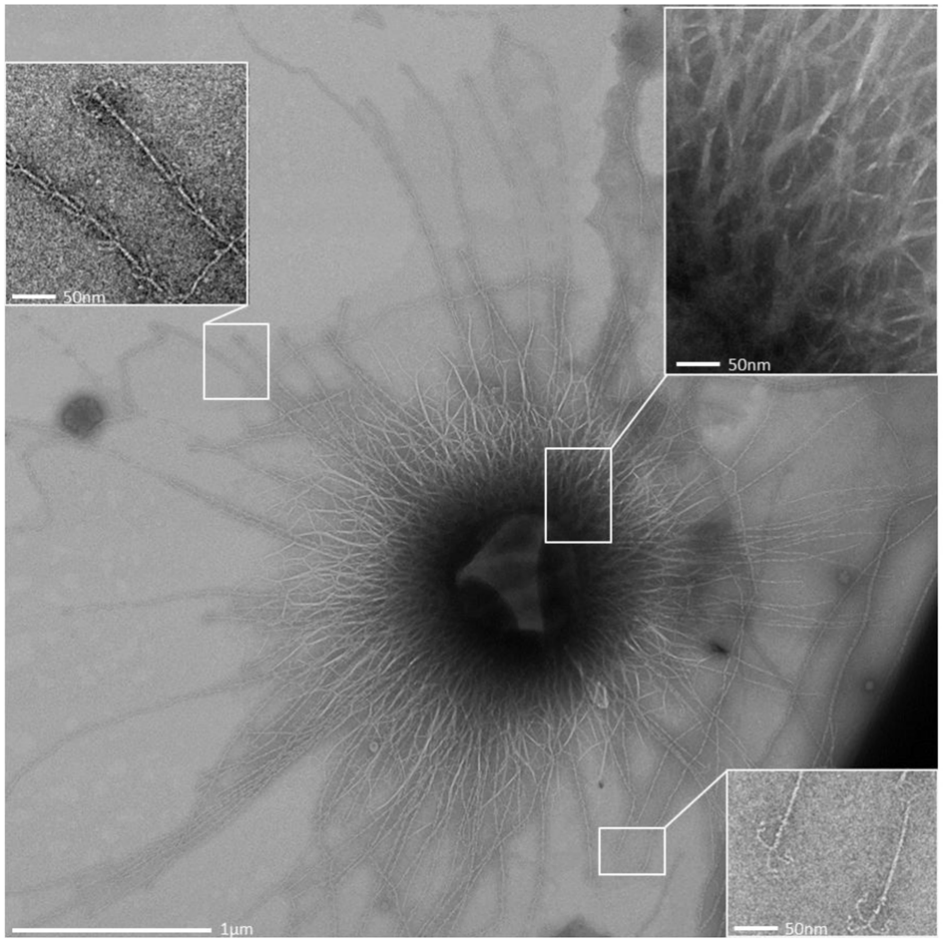
**Overview transmission electron micrograph (negative staining) of a SM1 euryarchaeal cell embedded in the EPS layer.** The architecture of the hami is shown in the close up views.

**Figure 6:**
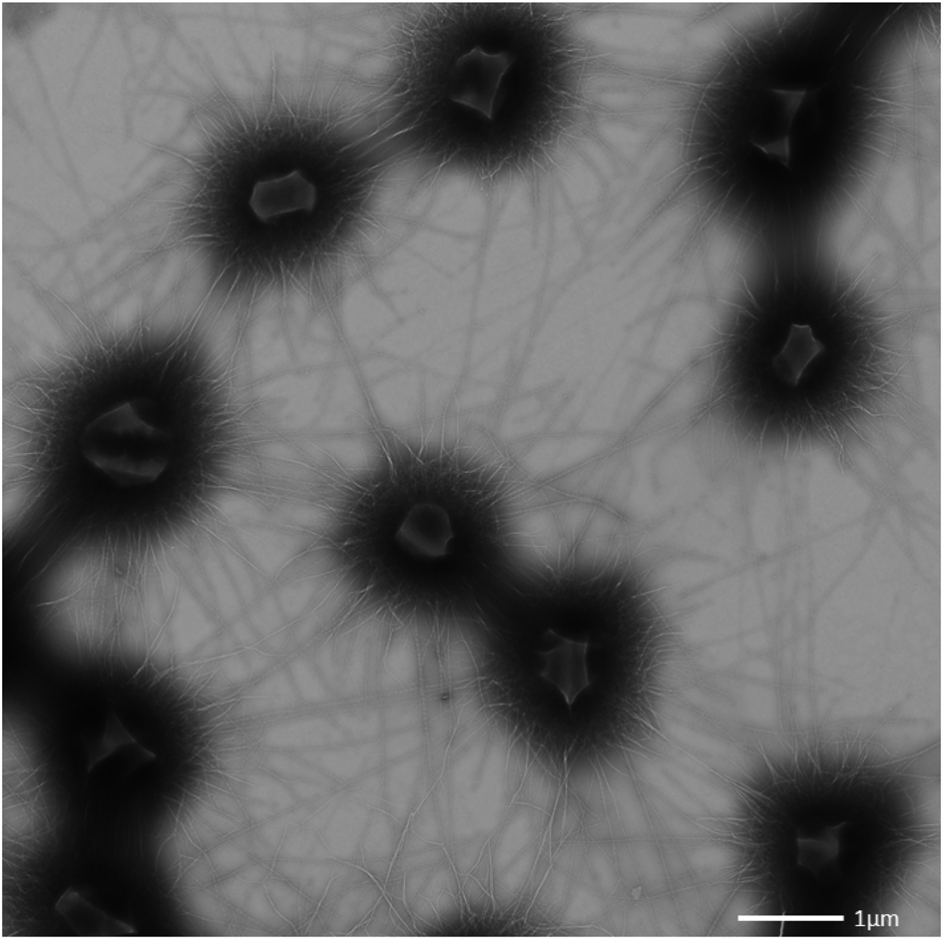
**Transmission electron micrograph (negative staining) of the SM1 euryarchaeal biofilm**.

The archaeal cells were arranged in mostly regular distances [~1.0–2.0 μm, mean: 1.26 μm, standard deviation (*SD*): 0.5 μm], forming a spacious, penetrable, but strongly connected cell-to-cell network (Figures 3A,B). Each cell within the biofilm was linked to 1–7 (mostly 6) cells by a dense web of cell-cell contact threads (Figures 3B and 1A). These connections occasionally appeared like tubes or bars (not shown), caused by drying artifacts due to a high amount of EPS, often covering the fine structures. This EPS layer resulted in the smooth appearance of cell surfaces and their surface appendages (Figure 3C). However, in different areas of the biofilm, where the EPS was thinner or absent, the fine-structures of cell-cell connections (the hami; Moissl et al., 2005) could be visualized in more detail (Figure 4). The EPS was shown to form a ~400 nm wide matrix around the cells (Figure 5). The hami protruded beyond the EPS, still allowing the cells to contact other cells or abiotic surfaces (Figure 6). In contrast to the regularly organized pattern between the archaeal cocci, bacteria did not have a certain distance to the archaea but were embedded in an irregular manner - they were either directly attached to an archaeal cell, located between several archaeal cells, or not attached to other cells at all (Figure 1), leading to the assumption that the interacting hami, and not the EPS, are the driving force to maintain the archaeal biofilm structure with defined cell-cell distances.

**Figure 7:**
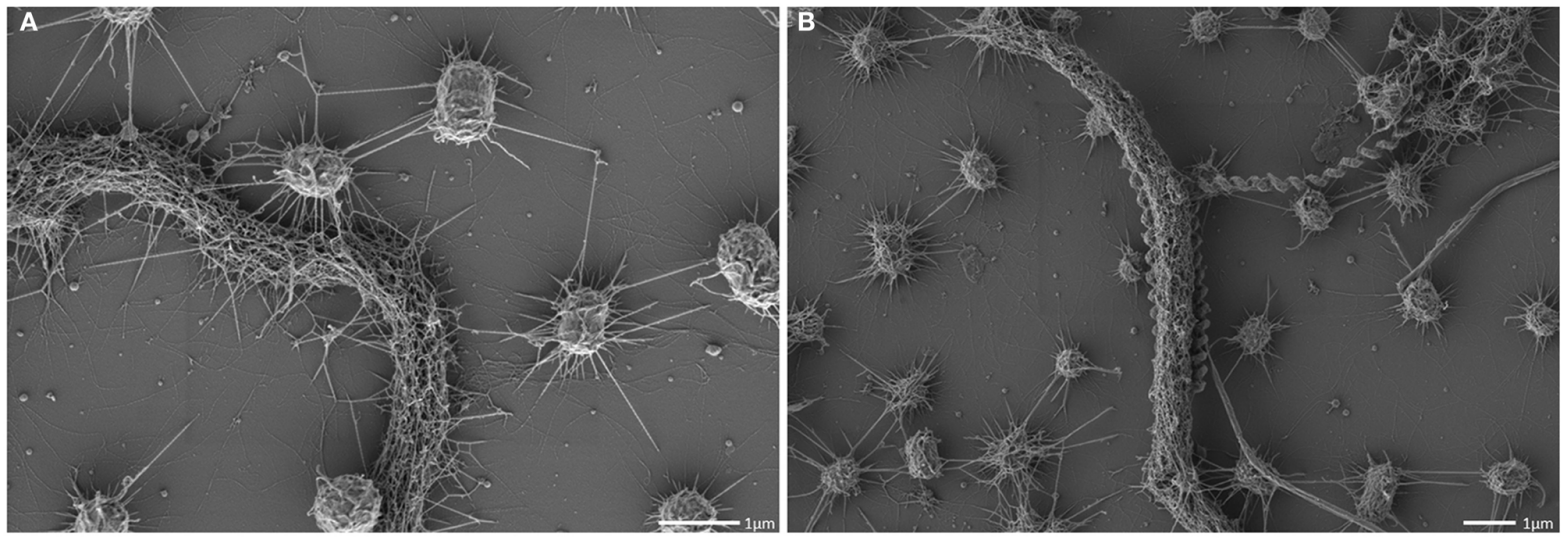
**Scanning electron micrograph.** Archaeal cocci of the SM1 biofilm with numerous hami, cocooning bacterial filaments of varying diameter **(A,B)**.

The interconnected coccoid archaea seemed to seek additional contact to bacterial cells (Figures 4, 7) via their hami. Noteworthy, some bacterial morphotypes (filament-forming rods) within the biofilm appeared to be cocooned by hami (Figure 7, Probst et al., 2014), whereas other bacteria (such as spirilla, Figure 7B) were only sparsely contacted.

### The SM1 euryarchaeal cell appendages: the hami

**Figure 8:**
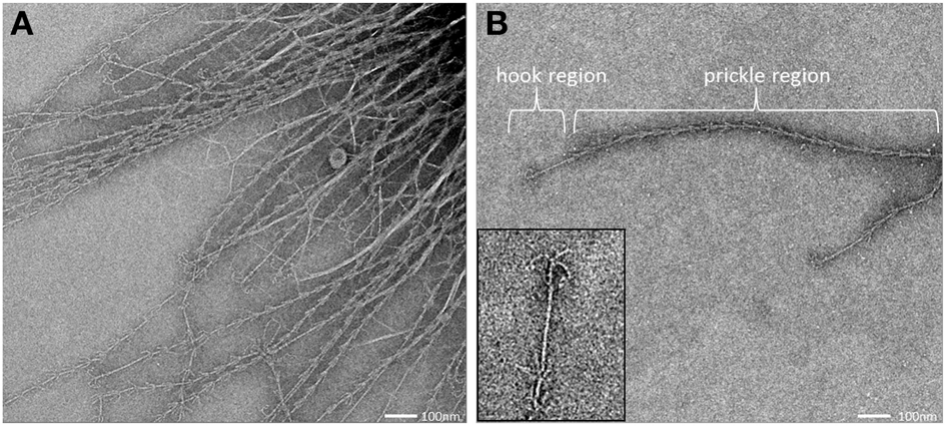
**Transmission electron micrograph (negative staining) of cell appendages (hami) protruding from a cell (A).** The hamus architecture is distinguishable into a prickle region and a hook region **(B)**, (see also Moissl et al., 2005).

All archaeal cells revealed the presence of hundreds of hami that protrude from their cell surfaces (Figures 5, 6, 8A). All hami analyzed (incl. TEM following negative-staining and unstained by cryo-TEM; Moissl et al., 2005) showed nano-grappling hooks at their distal ends (Figures 5, 8B). The hami architecture was clearly distinguishable in hook- and prickle-regions, where three prickles were formed in regular distances by the major filament (Figure 8B). These prickles are shaped by local bending of the three basic proteinaceous fibers (Moissl et al., 2005). The hooks were on average 60 nm in diameter (Figures 5, 8B) and were found to attach to the surfaces of other cells and to the prickle-regions or hooks of hami belonging to neighboring cells. The length of single hami was determined to be in the range of 0.4–3.7 μm, with an average length of 1.3 μm (*SD*: 0.6 μm).

### The SM1 euryarchaeal cell wall is composed of an inner and outer membrane

**Figure 9:**
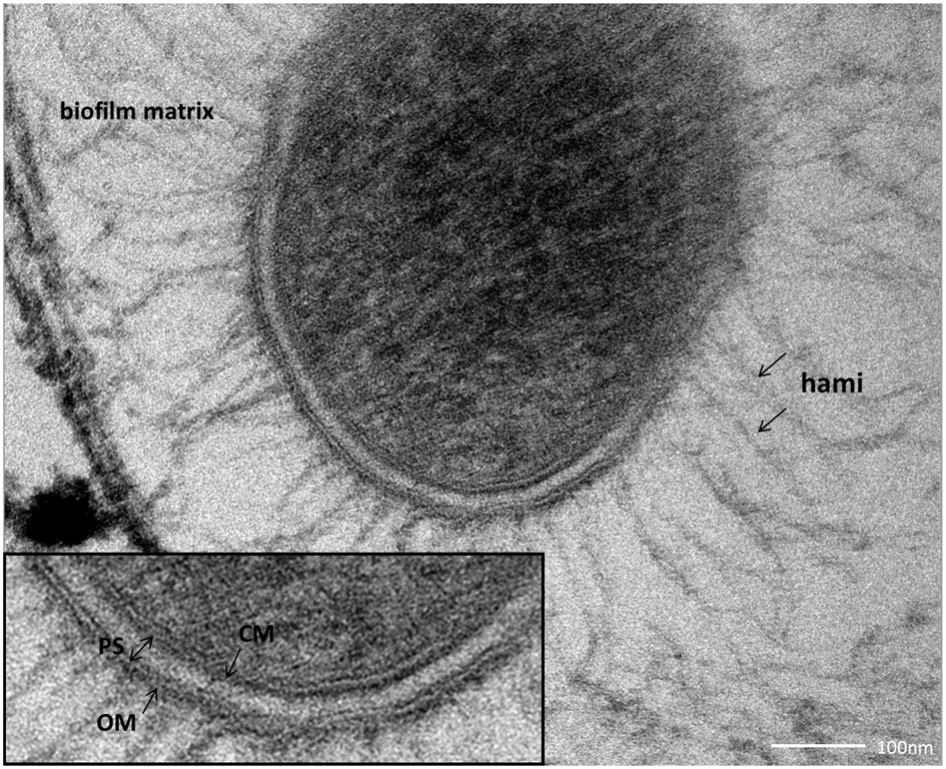
**Thin section of a single coccus, embedded in the biofilm matrix.** The close-up view reveals the clearly visible cellular membrane (CM), the periplasmic space (PS) and the outer membrane (OM).

**Figure 10:**
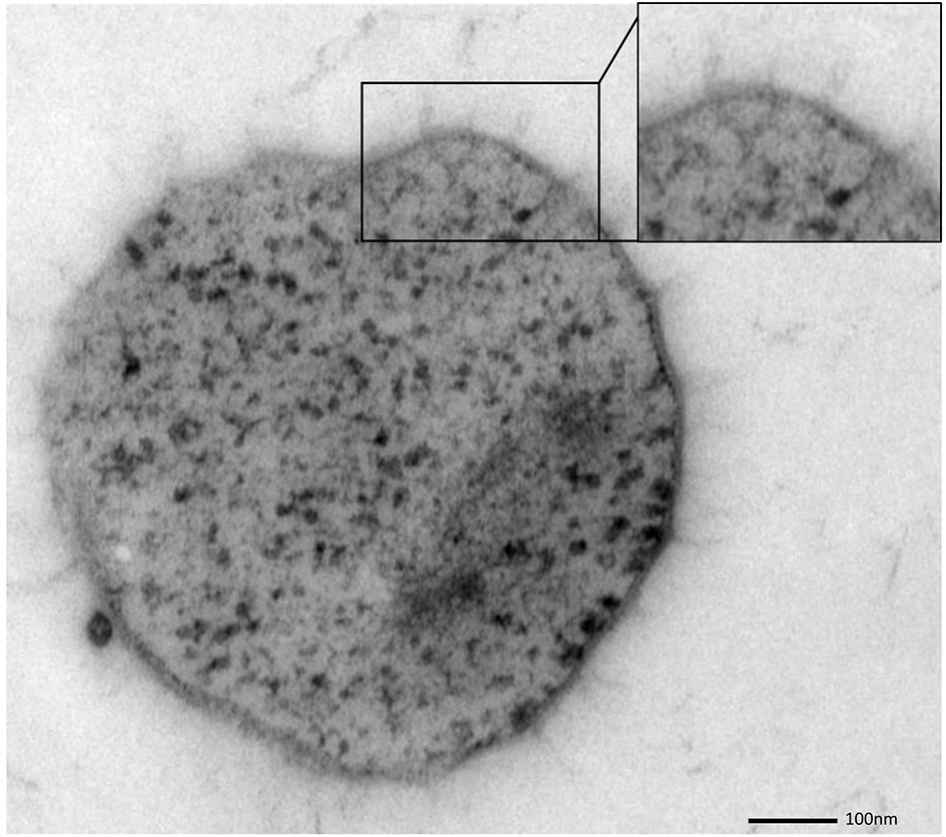
**Thin section of a single, coccoid, SM1 euryarchaeal cell.** The close-up view highlights several structures, possibly hami, which might cross both membranes.

SM biofilm samples were subjected to thin sectioning in order to analyze their ultrastructure in more detail. The outer sheath was identified as an additional membrane (Figure 9) and not, as often seen within the Archaea, as an S-layer. The SM1 euryarchaeal cell wall thus is composed of an inner membrane, periplasm, and an outer membrane. The inner and outer membranes revealed a typical structure (electron-dense, electron-lucent, electron-dense) and each showed an average thickness of 5–6 nm. The periplasm was determined to span 25 nm on average. The periplasm did not include any particles or other larger conglomerates or vesicles, as analyzed so far. Thin sections of cells further confirmed the presence of an EPS-layer and the hami forming a dense network around the cells (Figure 9). Although the anchorage of the hami could not be resolved so far, these filaments seemed to span both membranes (Figure 10). Within dividing cells, right at the central contraction site, belt-like structures were visible, suggesting protein aggregations involved in cell division machineries, such as FtsZ (Figure 2). The cytoplasm appeared packed with ribosomes and dark regions, which could display the chromosome or the location of storage substances (Figure 2).

## Discussion

The SM1 Euryarchaeon is a unique organism that shows many features not observed in other microorganisms. Its distinct position within the phylogenetic tree (Rudolph et al., 2004), the ability for biofilm-formation, and its predominance over associated bacteria, as well as the biofilms' origin in the subsurface of sulfidic springs warranted a detailed analysis of the ultrastructure. In this communication, we focused on the biofilms found in upwelling waters of sulfidic springs in the SM. Besides the discovery of the hami (Moissl et al., 2005), this current study provides the first detailed ultrastructural analyses of the SM biofilm population. Most of the knowledge about the SM1 euryarchaeal biofilms, however, was so far retrieved from the MSI environment (Henneberger et al., 2006; Probst et al., 2013, 2014), including preliminary ultrastructural insights (Henneberger et al., 2006).

The archaeal biofilm fine-structure appeared to be similar to described bacterial biofilm architecture (Sutherland, 2001), where the microbial cells are typically enclosed in a matrix of EPS (Costerton et al., 1995). Generally, this matrix is composed of DNA, proteins and polysaccharides and forms a slimy layer around the cells (Wingender et al., 1999). Data on the EPS composition of the SM SM1 biofilm are not available yet. DNA, however, was not detected in the highly hydrated MSI biofilm EPS, and the protein component was attributed to the presence of hami (Henneberger et al., 2006). Noteworthy, the amount of EPS was found to be variable: some cells were completely covered by EPS, whereas others were without detectable matrix.

In the bacterial domain, biofilm-formation is highly common and can cause severe problems in, e.g., medical environments (Donlan, 2001) or industrial facilities (Mattila-Sandholm and Wirtanen, 1992). On the other hand, biofilms are highly beneficial for food production or wastewater treatment (Park et al., 1990; Nicolella et al., 2000). EPS generally mediates the surface attachment, and forms a protection-shield against harmful chemical compounds (Bridier et al., 2011). Besides other important advantages, the biofilm matrix entraps excreted enzymes in close proximity to the cell (“external digestion system”; Flemming and Wingender, 2010). Water channels have been observed frequently in bacterial biofilms, which can support the distribution of nutrients and signal molecules, as well as the removal of inhibitory metabolic products (Costerton et al., 1994). The cells within the SM biofilms are organized in a strikingly regular pattern, in a spacious but strong and very sticky network, hinting at (1) a rapid flowing stream in its natural biotope in the subsurface, (2) the necessity of being attached to a surface, and (3) a requirement for a permanent water flow through the biofilm. Strikingly, compared to natural, non-medical bacterial biofilms, the purity and predominance of one species is extraordinary and was observed in both biofilms studied so far (Probst et al., 2014).

During the course of this analysis, numerous samples were taken from the sulfidic spring environment, transported under cool conditions and prepared for ultrastructural analyses as soon as possible. Due to the close vicinity of the two sampling sites to the Regensburg laboratory, transportation time was minimal (<1 h). However, due to the origin of the biofilms in the deeper subsurface of the sulfidic springs, which cannot be assessed at the moment, we have no information on the age or status of the biofilm pieces welled up with the spring water. In a previous study, the viability of the cells was found to be extraordinarily high (up to 90%), and cells exhibited excellent FISH (fluorescence *in situ* hybridization) signals due to the high content of ribosomes (Moissl et al., 2003), which are indications for a physiologically healthy status of the archaeal cells. Although precautions were taken in order to avoid preparation artifacts, caused by sampling or subsequent preparation for electron microscopy, alterations and damages cannot completely be avoided. This could be overcome by an immediate, on-site freezing of the samples for, e.g., cryo-electron tomographical analyses. This technique would allow for the detailed study of the cell division machinery, the hami anchorage, and the two-membrane system itself and thus is a desirable goal for subsequent studies.

All of the cells analyzed by electron microscopy carried about 150 hami on their surface, with an average length of 1.3 μm. This is within to the reported length-range of pili found on the surface of *Escherichia coli* (1.0–2.0 μm; Russell and Orndorff, 1992), which usually carries 100–300 pili (Neidhardt et al., 1990). Obviously the unique hami are well suited for the formation of such a biofilm, being responsible for cell-cell and cell-surface attachment. In addition, the hami, and in particular the prickle region, seem to facilitate the regular distance pattern, forming spacers between the cells (Moissl et al., 2005). Noteworthy, the SM biofilm cells were found to be significantly smaller than those of the MSI biofilms (0.60 μm vs. 0.72 μm; Probst et al., 2014). Based on SEM. the distances of SM cells to each other were 1.3 μm (on average), which is in strong contrast to confocal laser scanning microscopy data from the MSI population (4 μm distance). Currently it is unknown, whether this difference is based on strain-specific properties, or on method-specific preparation.

At this point of research, additional function(s) of the hami, besides attachment to surfaces, remain speculative. The energetic cost of hami synthesis appears higher than the production of simple, filamentous pili (which could also mediate surface adhesion), so that additional tasks might be envisaged. Thus, hami could be involved in cell motility, such as mediated by some bacterial type IV pili (Mattick, 2002; Ayers et al., 2010). Those can be retractile, and thus allow the bacterial cells to move on surfaces (“twitching motility,” Semmler et al., 1999; Maier, 2005). Although motility on a surface has not been observed for the SM1 Euryarchaeon so far, the cells might be able to control and regulate the attachment and the cell-cell distance via directed assembly and disassembly of the filaments. Another function could be electron-transfer, as observed for bacterial *Geobacter* species, which could allow cell-surface and cell-cell interactions (Reguera et al., 2005). Noteworthy, the SM1 Euryarchaeon seems to seek contact to bacteria of a specific morphotype: filament-forming, rod-shaped bacterial cells are frequently grappled by hami, and sometimes even completely cocooned by the surface appendages (see also Probst et al., 2014). This observation might pinpoint at a specific interspecies interaction (e.g., Näther et al., 2006; Fröls et al., 2008; Ajon et al., 2011; Bellack et al., 2011; Jarrell et al., 2011), but remains speculative at this point.

The SM1 Euryarchaeon possesses two membranes, which has rarely been described for Archaea. A typical archaeal cell wall is composed of a single membrane and an attached outer proteinaceous sheath (the S-layer), whose crystalline pattern can be used as a marker for certain genera and groups of Archaea (König et al., 2007; Rachel, 2010). It has been proposed that the S-layer represents the oldest cell wall structure (Albers and Meyer, 2011), since only few archaeal groups, such as several methanogens, members of the recently proposed *Methanomassiliicoccales* species [former classified as *Thermoplasmatales*, the seventh order of methanogens (Iino et al., 2012; Borrel et al., 2013)] and *Ignicoccus* species lack this protein layer. The latter possesses two membranes, where the outer cellular membrane (OCM) harbors the H_2_:sulfur oxidoreductase as well as the ATP synthase, and therefore appears to be energized (Küper et al., 2010; see Supplementary Figure S1). *Ignicoccus hospitalis* is in direct physical contact with its ectosymbiont/ectoparasite *Nanoarchaeum equitans*, which obtains several cell components from its host in order to compensate for its own biosynthetic shortcomings. The nano-sized archaeon is interacting with the host's OCM, facilitating the transport of amino acids, lipids and—although not experimentally proven yet—ATP molecules and cofactors in an yet unknown process (Huber et al., 2012). The unique cell architecture of all *Ignicoccus* species (Rachel et al., 2002; Junglas et al., 2008; Huber et al., 2012) in combination with the energized OCM demarcates *Ignicoccus* clearly from all known prokaryotic cell envelopes. To date, it is unknown whether the outer membranes of the Euryarchaeota *Methanomassiliicoccus* or SM1 are energized. This also remains unknown for the ultrasmall ARMAN cells, whose ultrastructure was interpreted as possessing an inner and OCM instead of an archaea-typical cell wall (Comolli et al., 2009; Baker et al., 2010). Except for the lipid composition, these membranes distantly resemble the dimensions and appearance of bacterial Gram-negative cell walls. It is not known whether such a cell wall architecture is rather a general feature of many archaea and was not recognized as such so far, or is an exception within this domain of life.

Strikingly, all archaea that possess a double membrane-based cell wall are involved in close interaction with other archaea, bacteria or their eukaryotic host. Bacteria which are participating in syntrophic partnerships are often found to be equipped with unique multiple membrane complexes (Orphan, 2009), and thus a positive effect on such interactions could be envisaged for several reasons: (1) An outer membrane is a suitable surface for anchoring proteins, lipids and carbohydrates, which could serve as contact sites for interactions (Mashburn-Warren et al., 2008). In contrast to S-layers, membrane architecture can be changed and regulated internally, allowing flexible responses to environmental changes. Within the SM1 Euryarchaeon, the double membrane also anchors the hami, which represent the major contact site of the cell toward biotic and abiotic surfaces. (2) The spanned periplasm provides additional space for metabolic products, chemosensors, signal cascades, storage compounds, and other molecules possibly involved in microbial interactions (Davidson et al., 1992; Wadhams and Armitage, 2004). Additionally two compartments provide the possibility of generating gradients and allow compartmentalization even within one single prokaryotic cell.

The finding of an increasing number of archaea with double-membrane cell walls could suggest this feature to be a general characteristic of a predecessor archaeon, and questions the S-layer as the (proposed) ancient cell wall type for Archaea. It shall be noted, however, that sample preparation and clear visualization of the undisrupted cell wall is challenging and, in most cases, has to include a careful interpretation of the obtained data. The question whether the double membrane is a general feature of Archaea emphasizes the need for more detailed ultrastructural analyses of cultivated and uncultivated archaea, but also asks the community to reconsider the proposed models for archaeal cell division and formation of cell surface appendages. The latter includes the involvement of other (novel?) translocation machineries for cell surface molecules, including the transfer across two membranes and the periplasm. Overall, it becomes again clear that the archaeal domain is not humble in structure, organization, and function. The more we learn about this group of microorganisms, the more we recognize the sophisticated, complex, and clever way of archaeal living.

### Conflict of interest statement

The authors declare that the research was conducted in the absence of any commercial or financial relationships that could be construed as a potential conflict of interest.
