# Supplementary material for: Grappling archaea: ultrastructural analyses of an uncultivated, cold-loving archaeon, and its biofilm
Source: Front Microbiol. 2014 Aug 5;5:397. doi: 10.3389/fmicb.2014.00397 (PMC4122167; doi:10.3389/fmicb.2014.00397)
Supplement: Supplementary file 1 [file Presentation1.PPTX]

## Slide 1
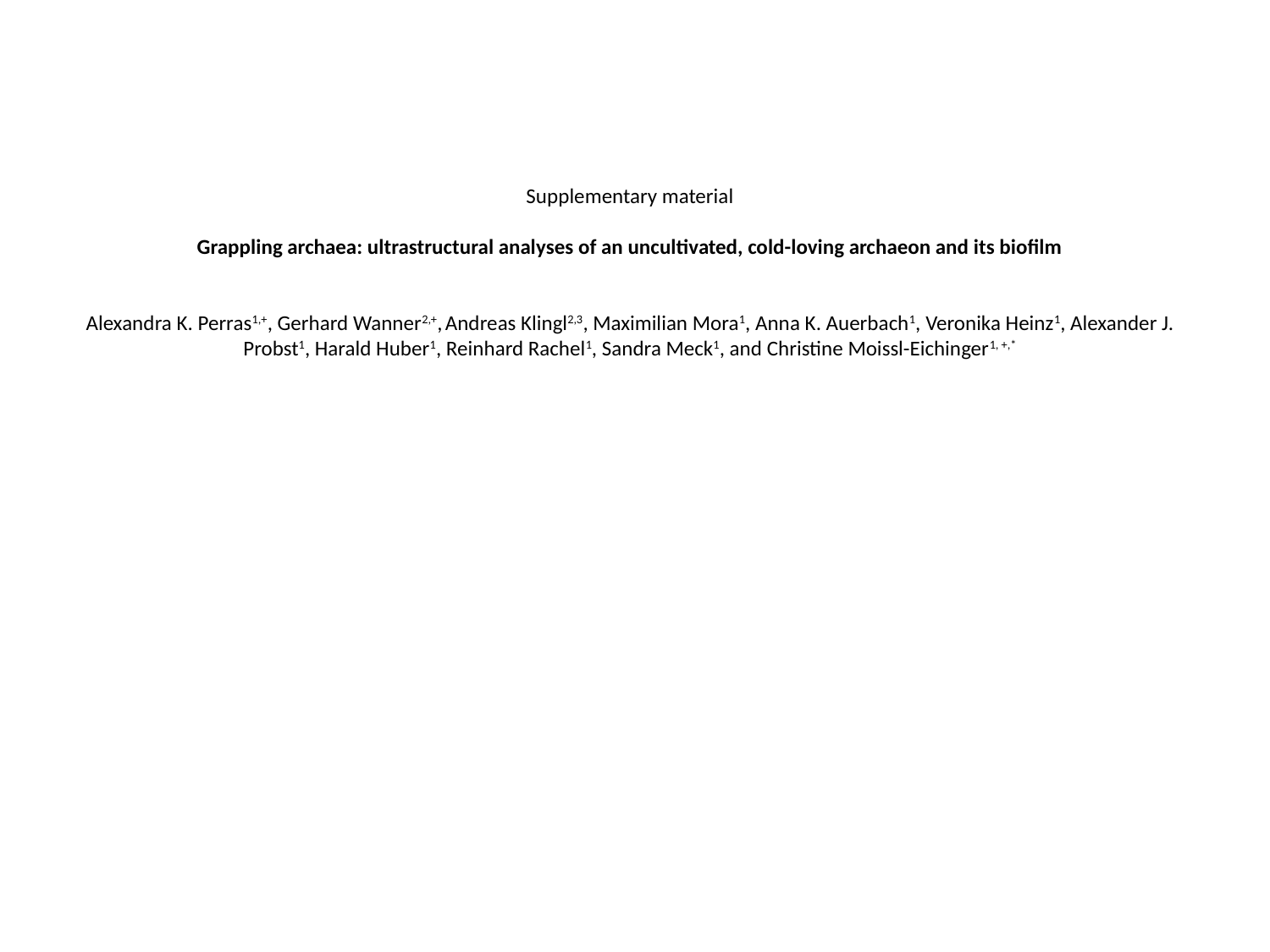

Supplementary material
Grappling archaea: ultrastructural analyses of an uncultivated, cold-loving archaeon and its biofilm
Alexandra K. Perras1,+, Gerhard Wanner2,+, Andreas Klingl2,3, Maximilian Mora1, Anna K. Auerbach1, Veronika Heinz1, Alexander J. Probst1, Harald Huber1, Reinhard Rachel1, Sandra Meck1, and Christine Moissl-Eichinger1, +,*

## Slide 2
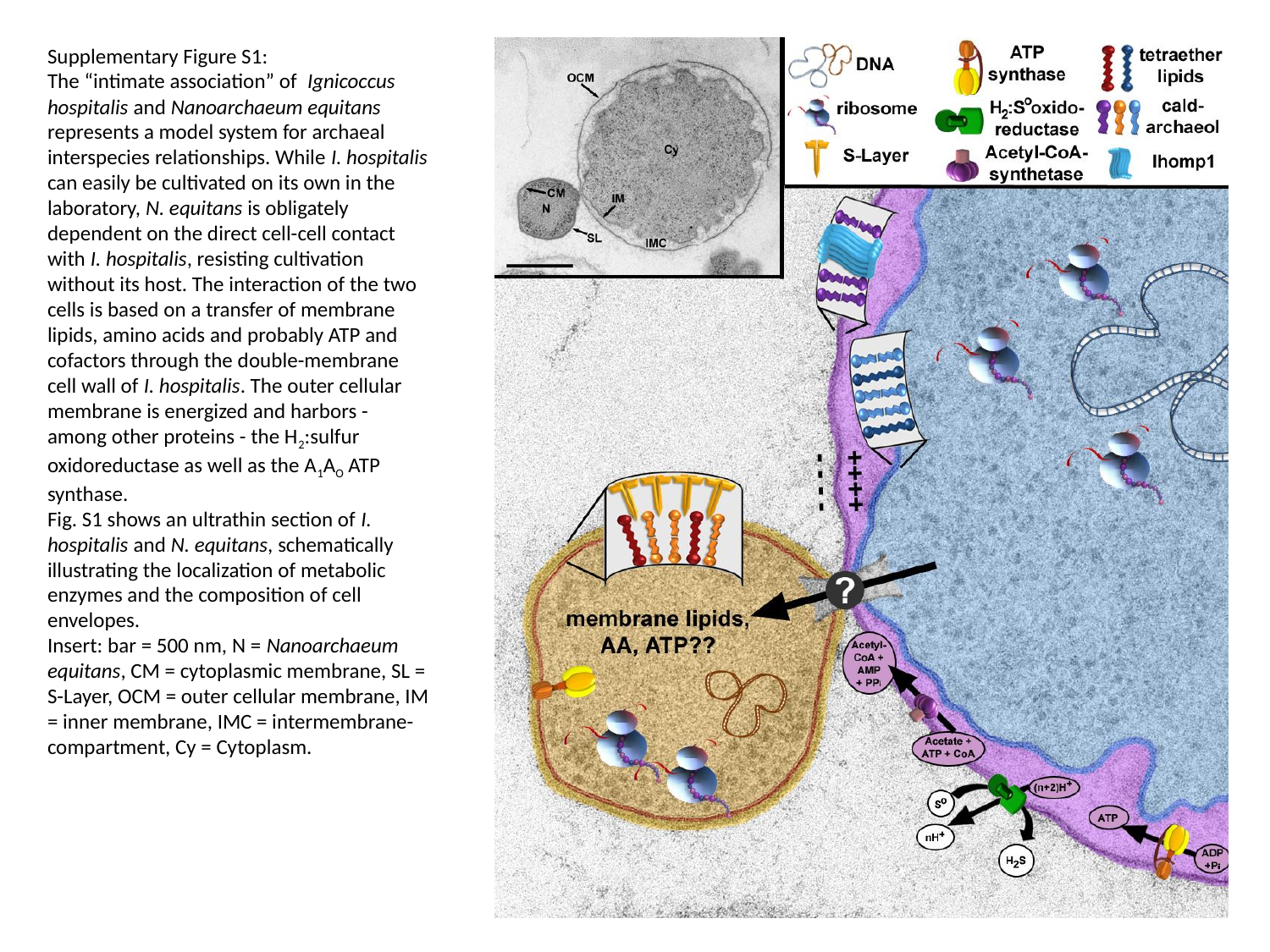

Supplementary Figure S1:
The “intimate association” of Ignicoccus hospitalis and Nanoarchaeum equitans represents a model system for archaeal interspecies relationships. While I. hospitalis can easily be cultivated on its own in the laboratory, N. equitans is obligately dependent on the direct cell-cell contact with I. hospitalis, resisting cultivation without its host. The interaction of the two cells is based on a transfer of membrane lipids, amino acids and probably ATP and cofactors through the double-membrane cell wall of I. hospitalis. The outer cellular membrane is energized and harbors - among other proteins - the H2:sulfur oxidoreductase as well as the A1AO ATP synthase.
Fig. S1 shows an ultrathin section of I. hospitalis and N. equitans, schematically illustrating the localization of metabolic enzymes and the composition of cell envelopes.
Insert: bar = 500 nm, N = Nanoarchaeum equitans, CM = cytoplasmic membrane, SL = S-Layer, OCM = outer cellular membrane, IM = inner membrane, IMC = intermembrane-compartment, Cy = Cytoplasm.
